# Technological Adjuncts to Streamline Patient Recruitment, Informed Consent, and Data Management Processes in Clinical Research: Observational Study

**DOI:** 10.2196/58628

**Published:** 2025-01-29

**Authors:** Jodie Koh, Stacey Caron, Amber N Watters, Mahesh Vaidyanathan, David Melnick, Alyssa Santi, Kenneth Hudson, Catherine Arguelles, Priyanka Mathur, Mozziyar Etemadi

**Affiliations:** 1 Kellogg School of Management Northwestern University Evanston, IL United States; 2 Northwestern Medicine Chicago, IL United States; 3 Feinberg School of Medicine Northwestern University Chicago, IL United States; 4 Department of Obstetrics and Gynecology Brigham and Women's Hospital Boston, MA United States; 5 Department of Obstetrics and Gynecology Massachusetts General Hospital Boston, MA United States

**Keywords:** digital health, patient recruitment, consent, technological adjuncts, data management, clinical research processes, automation, digital platforms, data warehouse, patient data, imaging data, pregnancy, clinical research methods

## Abstract

**Background:**

Patient recruitment and data management are laborious, resource-intensive aspects of clinical research that often dictate whether the successful completion of studies is possible. Technological advances present opportunities for streamlining these processes, thus improving completion rates for clinical research studies.

**Objective:**

This paper aims to demonstrate how technological adjuncts can enhance clinical research processes via automation and digital integration.

**Methods:**

Using one clinical research study as an example, we highlighted the use of technological adjuncts to automate and streamline research processes across various digital platforms, including a centralized database of electronic medical records (enterprise data warehouse [EDW]); a clinical research data management tool (REDCap [Research Electronic Data Capture]); and a locally managed, Health Insurance Portability and Accountability Act–compliant server. Eligible participants were identified through automated queries in the EDW, after which they received personalized email invitations with digital consent forms. After digital consent, patient data were transferred to a single Health Insurance Portability and Accountability Act–compliant server where each participant was assigned a unique QR code to facilitate data collection and integration. After the research study visit, data obtained were associated with existing electronic medical record data for each participant via a QR code system that collated participant consent, imaging data, and associated clinical data according to a unique examination ID.

**Results:**

Over a 19-month period, automated EDW queries identified 20,988 eligible patients, and 10,582 patients received personalized email invitations. In total, 1000 (9.45%) patients signed consents to participate in the study. Of the consented patients, 549 unique patients completed 779 study visits; some patients consented to the study at more than 1 time period during their pregnancy.

**Conclusions:**

Technological adjuncts in clinical research decrease human labor while increasing participant reach and minimizing disruptions to clinic operations. Automating portions of the clinical research process benefits clinical research efforts by expanding and optimizing participant reach while reducing the limitations of labor and time in completing research studies.

## Introduction

Clinical research processes, specifically in patient recruitment, are rife with logistical complexities that often determine the success or failure of a clinical trial [1]. Successful participant enrollment depends on organization, personnel, and participant factors as well as the features of the clinical trial at hand [2-5]. McDonald et al [6] cited reasons for research study failure as including heavy clinical and research workload of research team members, perceived imbalance between patient incentive and risk, and low status typically conferred to recruitment work. Of 114 studies in the review of McDonald et al [6], only 31% met their original recruitment goals, while 53% required extended periods for completion.

The resource-intensiveness of recruitment activities often dictates whether studies are completed successfully [7]. Recruitment processes have traditionally relied heavily on trained manual labor [8,9], which is costly. Moreover, these research personnel often face logistic barriers when recruiting in high-volume clinical settings, where their research activities may be restricted by clinical workflows and tight patient turnaround [10]. In general, in-person recruiting is personnel-intensive, as it requires research staff to repetitively explain the research study to each potential participant accurately and representatively [11,12]. Finally, traditional methods for recruiting patients rely on collecting and filing printed paperwork, especially in managing patient records and study results, which oftentimes require a high cognitive workload to manage and safeguard [2].

Following these observations, health care and federal institutions have increasingly demonstrated support for the integration of digital technological capabilities within clinical research [13-15], especially leveraging the capabilities brought about by automating clinical processes [16,17]. In 2019, the National Heart, Lung, and Blood Institute; National Institutes of Health; and National Science Foundation hosted a workshop calling for the digitization of clinical research using advanced analytics to facilitate patient screening and data management to increase the representation of diverse patient populations [18]. In a separate review of recruitment methods across 61 clinical trials, digital recruitment was found to be 52% more effective than conventional offline recruitment [19].

Such technological advancements present opportunities for overcoming the onerous complexities of clinical research workflows while also reducing the operational costs of research (by reducing research staff labor-intensiveness and expanding potential participant reach) as well as the potential for human error in recruitment processes (by digitizing data management). In this paper, we share our experience using technological adjuncts to streamline participant identification, recruitment, consent, and data management processes for an observational clinical research study. These strategies allowed us to complete our study recruitment goal ahead of schedule and in a cost-effective, resource-optimized way.

## Methods

### Ethical Considerations

All elements of the research study—which, in this portion, involved patient recruitment to achieve the goals of our broader study—were approved by Northwestern University’s Institutional Review Board Office (study ID: STU00215717). This approval confirms that all aspects of the study meet the ethical standards of the review board. Invited patients were asked to provide consent if they agreed to participate in our study and were also given the option to opt out of the study with no consequences to the care provided to them. Patients were not financially compensated for their participation in our study, and all data collected were subsequently deidentified.

### Purpose of Broader Research Study

The digitized recruitment processes that we describe in this paper are part of a prospective, observational research study. The research study’s primary aim is to develop a database of ultrasonography images for use in the development of artificial intelligence technology with specific applications to perinatal care. To fulfill this goal, we planned to enroll 1000 patients across various time periods of pregnancy from within a single institution. To accomplish this task, we designed and executed a digitized process of patient recruitment and data management.

**Figure 1:**
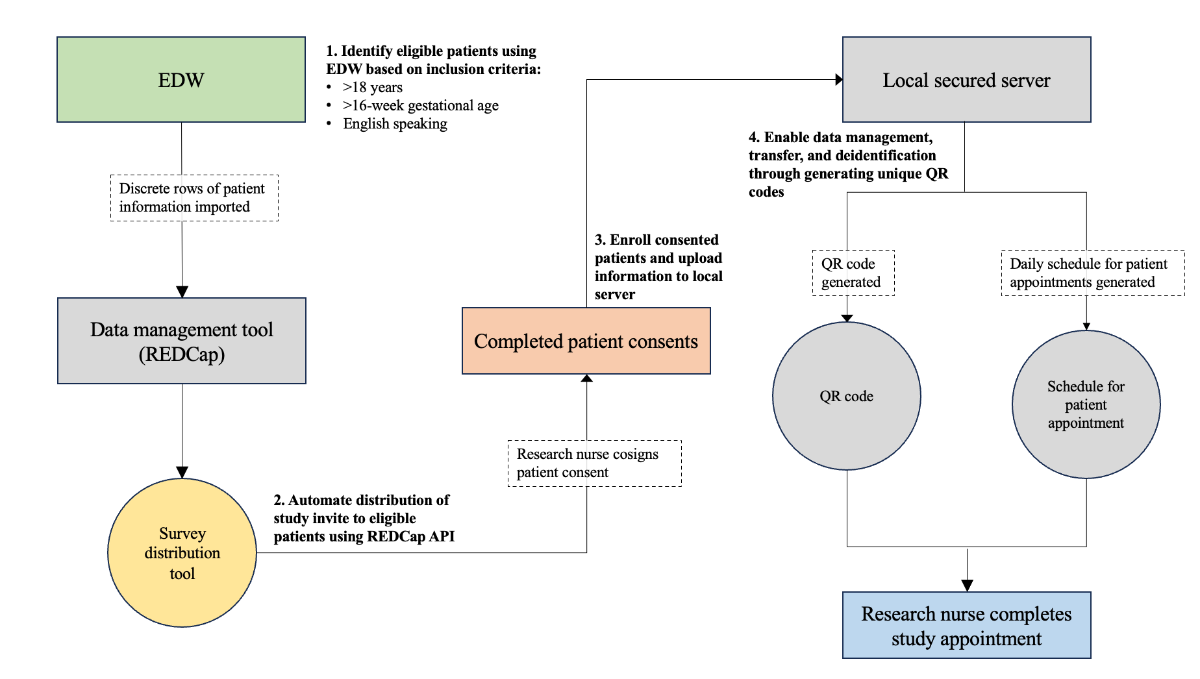
Workflow for clinical research processes across digital adjuncts. API: application programming interface; EDW: enterprise data warehouse; REDCap: Research Electronic Data Capture.

Given our ultimate goal to build algorithms capable of providing diagnostic recommendations and predictions of various maternal and fetal health outcomes, we engaged in strategic and targeted patient recruitment to ensure that we recruited an appropriate number of patients with specific health conditions across various outcomes while abiding by constraints including a temporally bounded funding structure. Our clinical research processes relied on 3 main technological adjuncts: a secured, centralized database of electronic medical records (enterprise data warehouse [EDW]), a research data management tool (REDCap [Research Electronic Data Capture]; Vanderbilt University), and a locally managed, Health Insurance Portability and Accountability Act–compliant QR code–based system that allowed us to coordinate all patient information, data, and enrollment-related documentation on a single, secured server. An overview of our clinical research processes is depicted in Figure 1. Each step of these processes is detailed below.

### Identify Eligible Patients Using EDW Based on Inclusion Criteria

The patient recruitment process leveraged access to patient medical records collated from Epic and stored in a local EDW to identify eligible patients for the study. In doing so, we performed a SQL query, filtering for patients based on the inclusion and exclusion criteria and limited to participating clinical units. The inclusion criteria were patients aged 18 years and older, English speaking, and at least 16 weeks of gestational age. This query derived multiple rows of eligible patients to be invited to participate in our study. We set up this query function as an automated process with the list of eligible patients refreshed every 24 hours. This query generated a list of patient appointments that had been scheduled within the next 30 days across participating clinics.

### Automate Distribution of Study Invite to Eligible Patients Using the REDCap Application Programming Interface

We used a REDCap-specific application programming interface to import the rows of eligible patient appointments generated from the EDW into the REDCap database. Each discrete row provided all necessary details for patient identification and recruitment including patient medical record numbers, personal and clinical information, contact information, and details including patient notes on upcoming appointments. Patients provide universal consent when first receiving care at our institution, thereby granting access to contact information for use in recruitment for research studies.

Within REDCap, we conducted an additional manual check on the list of eligible patients against our inclusion criteria using a filtering function. This was done to mitigate any errors that may have been made as a result of data compatibility issues during transfer between platforms. Given that patients are exclusively electronically recruited for this study, we also filtered patients based on the presence of an email address on file. Finally, we filtered to exclude patients who previously indicated disinterest in participating in this study, resulting in a narrowed list of eligible patients to recruit.

We used REDCap’s survey distribution tool function to distribute invitations to eligible patients. We sent personalized emails with patient names as designated in their charts followed by all relevant information pertaining to the study including the title and purpose of clinical research, risks and benefits of participation, participant expectations for the study, and contact information for any inquiries. Patients were provided unique links that directed them to their personalized electronic consent forms embedded in REDCap. The email invitation text may be reviewed in Multimedia Appendix 1.

### Enroll Consented Patients and Upload Information to Local Server

As the research nurse on the team cosigned patient consent forms, using a second SQL query, we uploaded the list of consented patients onto a local server managed by the project team. On the server, expired patient appointments were removed to optimize memory space. With the list of consented patients, we deployed a third SQL script that generated a list of patients who had an appointment in the clinical units each day. This script also generated a unique QR code for each patient to associate the collected data with the patient’s medical records on the local server and in REDCap.

With the list of consented patients, the research nurse scheduled data collection appointments by prioritizing patients with outcomes of interest to our study, and also inconsideration of where each patient was located and the providers’ preference for data collection should be carried out. Scheduling accounted for travel time between appointment rooms and clinical units and the time taken to prepare for and wrap up data collection. This routine enabled the research nurse to carry data collection in multiple clinical units in a single day.

### Enable Data Management, Transfer, and Deidentification Through Generating Unique QR Codes

As is standard for most data collection studies, the study workflow must culminate in generating a fully deidentified database. Alongside generating the QR code, a unique research examination ID that is not present in the patient’s medical record is generated as a function for identifying collected data without associating it with any protected health information (PHI).

At the beginning of each data collection appointment, a research nurse would scan the unique QR code that would route the collected ultrasound scans to the appropriate folder on the local server to be stored. The folders on the server are labeled using the unique examination ID. This system allows the QR code to serve as a means for identification and reference as we link deidentified patient files with the associated ultrasound scans. Aside from a single “master list” linking the patient’s “real” PHI information to their study information (as is standard in data collection studies), routing and parsing relevant data points are fully automated and rely on research-specific identifiers (unique examination ID) and QR codes, thus reducing the risk of study documentation being lost or otherwise mishandled. This contrasts current, manual approaches that oftentimes rely on names or date of births written on documents to associate data to a patient, which subsequently must be shredded and disposed of.

## Results

Between December 1, 2021, and June 13, 2023 (19 months), we achieved our study goal of enrolling 1000 patients for the study. Our automated EDW queries identified 20,988 eligible patient appointments across 3 obstetric clinics throughout that time period. Each query was automatically run every 24 hours and generated between 1000 and 2000 eligible patient appointments.

Logistic constraints including limited study personnel (1 research nurse on the project) and limited work schedule availability to collect data resulted in only approximately 50.4% (10,582/20,988) of identified eligible patients receiving an invitation to participate in the study. We also restricted invites to 2 or 3 potential patients with overlapping appointment times across all clinical sites. Data collection appointments were only scheduled on weekdays between 8 AM and 4 PM.

Based on these constraints, personalized email invitations were subsequently issued to 10,582 potential participants. We enrolled 1000 patients, yielding a 9.5% enrollment rate over 19 months. Among those who were enrolled in the study, 779 patients completed the research study visit within the reported time frame. The difference between enrolled patients and those with complete data collection is a result of some consented patients not having completed their study appointments within our reported time frame. This is largely attributed to the rescheduling of data collection to a subsequent appointment to accommodate the time or logistic constraints of our 1 research nurse performing study data collection. During the study interval reported here, a total of 549 unique individual patients participated in the study; some patients participated more than once during their pregnancy.

**Figure 2:**
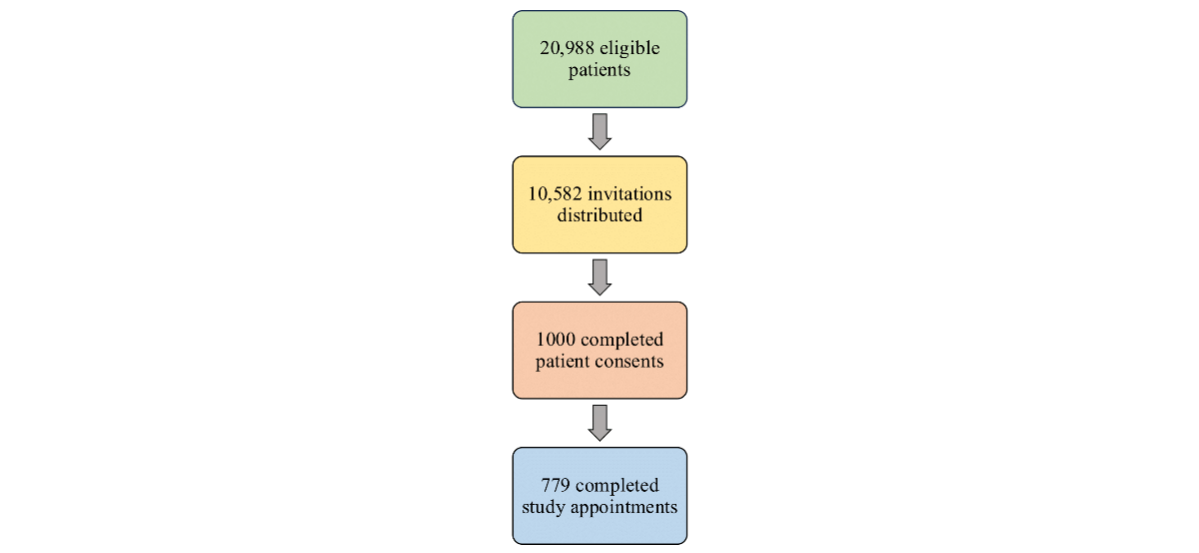
Overview of patient recruitment data across the recruitment and enrollment process.

Table 1 presents the descriptive demographics of participating patients for race, ethnicity, age, gestational age, and insurance payor. The patient recruitment and enrollment process are summarized in Figure 2.

**Table 1:** Demographic data of the consented patient population at the time of study enrollment.

			Study population (raw), n (%)		Institution population (raw), n (%)
**Race**					
	Asian	44 (8.0)		3958 (7.2)	
	Black	74 (13.5)		5452 (9.9)	
	White	284 (51.7)		35,930 (65.1)	
	Other	40 (7.3)		3363 (6.1)	
	Not specified	107 (19.5)		8028 (14.5)	
**Ethnicity**					
	Hispanic	80 (14.6)		11,403 (20.6)	
	Non-Hispanic	396 (72.1)		40,665 (73.6)	
	Not specified	73 (13.3)		3160 (5.7)	
**Age group (years)**					
	<20	4 (0.7)		1309 (2.4)	
	20-30	101 (18.4)		16,770 (30.4)	
	30-40	404 (73.6)		34,474 (62.4)	
	>40	40 (7.3)		2675 (4.8)	
**Gestational age (years)**					
	<16	2 (0.3)		794 (2.8)	
	16-20	167 (16.7)		3781 (18.2)	
	21-25	163 (16.3)		2666 (12.9)	
	26-30	196 (19.6)		3120 (15)	
	31-35	315 (31.5)		5502 (26.5)	
	36-40	157 (15.7)		4693 (22.6)	
	>40	0 (0)		183 (0.8)	
**Insurance payor**					
	Public	126 (23)		1286 (33.1)	
	Private	423 (77)		2595 (66.9)	

## Discussion

### Principal Findings

Overall, relying on digital adjuncts in this study helped us to achieve our patient enrollment goal in less than 19 months (as compared to an initial projection of 24 months) while optimizing operational costs and minimizing disruptions to participating clinical sites. For this study, using SQL automation within the EDW allowed us to accurately identify and regularly update lists of eligible participants without ongoing human input or intervention. Digitizing recruitment and consent expanded our reach to many more eligible participants than 1 research nurse could have realistically approached via in-person recruitment, thereby shifting the research nurse’s efforts and availability to collecting ultrasound scans and ensuring a positive patient experience participating in the study. Finally, using a digital interface simplified and contained data management for the study to a single server, which reduced the human burden of data management and consequently improved data integrity.

### Strengths

Consistent with the existing literature, we found digitizing clinical research processes to be effective in attaining goal recruitment rates, minimizing disruptions to clinic workflows, and ensuring transparency of information regarding research studies [9,19,20]. We also experienced the benefits of integrating the technological capabilities of the electronic medical record for broad-based identification of eligible participants [21-23].

Digitizing patient recruitment and enrollment allowed us the flexibility to strategically target patients with specific outcomes or appointments in specific locations. This also meant that our recruitment data were available to be analyzed frequently, thereby providing us with real-time feedback to improve our recruitment methods. Based on this feedback, we adjusted the time interval between sending an invitation and the scheduled appointment date to optimize participant consent rates.

Reducing aspects of clinical research that are highly dependent on human labor helped to alleviate financial constraints that have previously crippled clinical research and instead focused efforts on participant experience throughout the study [24]. Over 19 months of active patient recruitment with only 1 research nurse, our approach to patient recruitment enabled the research nurse to successfully collect data from patients across 3 clinical units that were in close geographic proximity, completing data collection appointments with up to 5 participants per day. In comparison, a study looking at consent rates using traditional in-person recruitment found that 9 recruiters were needed to approach 2498 patients over a 1-year period in a primary care physician office [24]. If we extended those ratios to our study, we would have needed 18 recruiters over 2 years to approach the 10,582 patients we reached by personalized email invitation.

In clinic settings where multiple clinical research studies are conducted concurrently, front-loading recruitment activities and consent limited disruption to the clinic workflows and minimized patient recruitment fatigue or coercion. We found that limiting research personnel presence in clinics with high volumes of patients and quick patient turnaround increased clinics’ willingness to collaborate as recruitment sites for our study.

For patients, shifting recruitment processes onto digital platforms allowed them unlimited time to review holistically all the information pertaining to the study, their participation in the study, and address any data privacy concerns before agreeing to participate in our study. Digitizing patient recruitment inherently standardized the information shared with all eligible participants, giving us confidence in the ethical execution of informed consent.

Finally, our QR code system alleviated pressures on research nurses to maintain cumbersome paperwork associated with data management and instead focus on collecting data and the patient’s experience in the study. Additionally, digitizing data management leveraged the compatibility between already digitized patient information (on Epic) and routed relevant study data, thus simplifying the deidentification process of PHI, especially when sharing data with external parties.

### Limitations

A limitation of patient recruitment via digital platforms is the potential for selection bias, in our case by restricting recruitment to patients with an associated email address. To mitigate this, research teams may wish to complement digital recruitment activities with telephone or in-person outreach to directly target groups at risk of exclusion.

We recognize that our study was minimally invasive and posed no risk to participants. As such, our results using technological adjuncts for clinical research processes may not be generalizable to clinical research with different study features. Additionally, the scope of this study did not include patient feedback on the recruitment process, and patient experiences with digital recruitment processes are a valuable area for future consideration.

Finally, using digital adjuncts in clinical research inherently means that research teams must include team members well-versed in the technical skills and know-how for setting up digitized recruitment systems and troubleshooting when needed. This option may not be readily available for all research programs or may require consideration of the costs of such expertise.

### Conclusions

Digital adjuncts are promising tools that can assist in streamlining patient identification, recruitment, and enrollment in clinical research studies. In our experience, digital adjuncts allowed us to reach out to many more patients than would have been possible with traditional one-on-one, in-person–based recruitment. Additionally, we believe that the benefit of privately reviewing information pertaining to the study without time constraints or any in-person influences optimizes an ethical consent process for patients. Finally, streamlining the data management necessary for our imaging-based observational study has reduced the personnel and resources required for the completion of the study. Using these digital tools could ease research and make it more equitable, leading to higher probabilities of study completion.

## Appendix

Multimedia Appendix 1 Personalized invitation sent to patients to participate in study.

## Abbreviations

EDW: enterprise data warehouse
PHI: protected health information
REDCap: Research Electronic Data Capture
