# Supplementary material for: Technological Adjuncts to Streamline Patient Recruitment, Informed Consent, and Data Management Processes in Clinical Research: Observational Study
Source: JMIR Form Res. 2025 Jan 29;9:e58628. doi: 10.2196/58628 (PMC11822312; doi:10.2196/58628)
Supplement: Multimedia Appendix 1 [file formative_v9i1e58628_app1.docx]

Sample Recruitment Letter

Dear [patient name as found on EDW],

You are scheduled for an obstetric appointment with [clinic name], which qualifies you for a research study focused on prenatal ultrasound.

The goal of the study is to collect ultrasounds from pregnant women to create a ‘data pool’. This collection of data will then be used to develop software that does not require a trained operator to capture ultrasound images. This will increase access to ultrasound for pregnant patients in areas of the world where ultrasound technology is not readily available.

The study is [title]. For more details about the study, please click or copy and paste the following link, [link to study].

If you are interested in participating, **please complete AND submit the consent up to the evening before your appointment**. Once consented, I will meet with you immediately before or after your OB appointment. We will then use the ultrasound(s) to look at your little one. This is a non-medical ultrasound and is not a part of your OB care.

Your participation is completely optional and voluntary. More detailed information about this study is within the informed consent form, available here:

[survey-link]

If the link above does not work, try copying the link below into your web browser: [survey-url]

This link is unique to you and should not be forwarded to others.

If you do not wish to be contacted about this study or have questions, you may notify the study staff at the email address listed below.

Thank you for considering participation in this impactful research study!

[Research Nurse sign-off]
